# Determination of the Time Window of Event-Related Potential Using Multiple-Set Consensus Clustering

**DOI:** 10.3389/fnins.2020.521595

**Published:** 2020-10-21

**Authors:** Reza Mahini, Yansong Li, Weiyan Ding, Rao Fu, Tapani Ristaniemi, Asoke K. Nandi, Guoliang Chen, Fengyu Cong

**Affiliations:** ^1^School of Biomedical Engineering, Faculty of Electronic Information and Electrical Engineering, Dalian University of Technology, Dalian, China; ^2^Faculty of Information Technology, University of Jyvaskyla, Jyvaskyla, Finland; ^3^Reward, Competition and Social Neuroscience Lab, Department of Psychology, School of Social and Behavioral Sciences, Nanjing University, Nanjing, China; ^4^Institute for Brain Sciences, Nanjing University, Nanjing, China; ^5^Department of Psychiatry, Chinese PLA 967th Hospital, Dalian, China; ^6^Department of Electronic and Computer Engineering, Brunel University London, Uxbridge, United Kingdom; ^7^School of Artificial Intelligence, Faculty of Electronic Information and Electrical Engineering, Dalian University of Technology, Dalian, China; ^8^Key Laboratory of Integrated Circuit and Biomedical Electronic System, Liaoning Province, Dalian University of Technology, Dalian, China

**Keywords:** multi-set consensus clustering, time window, event-related potentials, microstates analysis, cognitive neuroscience

## Abstract

Clustering is a promising tool for grouping the sequence of similar time-points aimed to identify the attention blocks in spatiotemporal event-related potentials (ERPs) analysis. It is most likely to elicit the appropriate time window for ERP of interest if a suitable clustering method is applied to spatiotemporal ERP. However, how to reliably estimate a proper time window from entire individual subjects’ data is still challenging. In this study, we developed a novel multiset consensus clustering method in which several clustering results of multiple subjects were combined to retrieve the best fitted clustering for all the subjects within a group. Then, the obtained clustering was processed by a newly proposed time-window detection method to determine the most suitable time window for identifying the ERP of interest in each condition/group. Applying the proposed method to the simulated ERP data and real data indicated that the brain responses from the individual subjects can be collected to determine a reliable time window for different conditions/groups. Our results revealed more precise time windows to identify N2 and P3 components in the simulated data compared to the state-of-the-art methods. Additionally, our proposed method achieved more robust performance and outperformed statistical analysis results in the real data for N300 and prospective positivity components. To conclude, the proposed method successfully estimates the time window for ERP of interest by processing the individual data, offering new venues for spatiotemporal ERP processing.

## Introduction

The event-related potentials (ERPs) carry important information about the cognitive process evoked by the brain response in milliseconds of the temporal domain. Almost all the ERP components are influenced by the attention corresponding to the latencies from the individual and a group of subjects (Luck and Kappenman, 2012). The latencies of ERP components can be considered as a stable brain electric field configuration (topography map) in milliseconds associated with the specific psychological process (i.e., attention module) (Lehmann, 1990). Moreover, measuring the ERP of interest undertakes a fundamental role in identifying and interpreting the cognitive process in the experiment. The most common approach to measure the magnitude and timing of the ERP of interest is to investigate the amplitude and the latency of peak voltage in the experimentally defined time window. Thereby, an important issue in the analysis of ERPs is how to define or select time windows. This influences both identifying components and performing statistical analyses. Hence, if the time window is not appropriately defined, the comparison between different conditions/groups can lead to unreliable and wrong psychological interpretations (Luck and Gaspelin, 2017).

The traditional ERP approach is to obtain the mean of measured potentials over a fixed and/or experimenter defined time window. The assumption is that the brain electric field configuration is stable for different conditions/groups, although this assumption is not empirically verified. Apart from widely used conventional ERP techniques such as latency peak and mean amplitude, numerous studies have used moving time-window technique and high-resolution time-bin analysis (e.g., each 5 ms) for measuring the peak (Van Overwalle et al., 2009; Mu and Han, 2010; Wills et al., 2014). Although moving time-window or point-by-point analysis in spatiotemporal ERP can provide more fine-grained temporal characterization and significant statistical results (Rotshtein et al., 2010), they can dramatically increase the probability of reporting errors (Luck and Gaspelin, 2017). In the above reviewed methods, the variety of responses, which dynamically influence the duration of time windows in different conditions/groups, are neglected.

Another group of researchers investigated the brain response states by analyzing the topographical changes (Lehmann, 1989, 1990; Lehmann et al., 1994; Micah et al., 2009) to determine the components of interest. The underlying assumption is that the electric field configuration does not change randomly as a function of time, despite exhibiting stability for tens to hundreds of milliseconds involving intervals of topographic instability (Lehmann et al., 1987; Murray et al., 2008). The clustering of spatiotemporal electroencephalogram (EEG)/ERP was used to capture template maps (i.e., topographies found by the clustering) which identifies the recorded signal (Lehmann, 1989, 1990). Hypothetically, the brain state (i.e., the brain electric field configuration) does not change during a specific response time (Lehmann, 1990; Pascual-Marqui et al., 1995; Lehmann et al., 2009). Consequently, the spatial correlation of corresponding topographies of the time-points in the cluster map is close to 1 (Pourtois et al., 2008). Two clustering algorithms in EEG/ERP research, namely, modified *k*-means (Pascual-Marqui et al., 1995) and agglomerate hierarchical clustering (AAHC; Tibshirani and Walther, 2005; Murray et al., 2008) were predominantly used in EEG/ERP researches. Two global measurements together, namely, global field power (GFP) and the global map dissimilarity (GMD), and the global explained variance (GEV) of the template maps (the most important cluster maps), for quantifying the template maps, were applied. Furthermore, the topographical analysis for spatiotemporal ERPs using clustering methods has been explored in several studies (Murray et al., 2008; Micah et al., 2009; Koenig et al., 2014). So far in the aforementioned microstates analysis studies (Michel and Koenig, 2018), determination of template cluster maps with higher explained variance and *post hoc* determination of microstates by fitting those maps to the data (topography maps) were used. As a result, the time-points are clustered based on their similarity in the electrode field configuration. Alternative methods, for cluster or factor analysis, such as optimized *k*-means with genetic algorithm and principal component analysis (PCA) (Williams et al., 2015), topographic pattern analysis, and PCA in high-density ERP (Pourtois et al., 2008) were utilized to determine the most dominant spatial components from the map series. Although independent component analysis and PCA are standard methods and are used for decomposition of the EEG/ERP with cluster analysis, the determination of the event of the interest is subjective instead of being the objective exploration of ERP.

Importantly, finding the suitable time window for measuring the ERP of interest using microstates analysis has also been studied in the numerous literature (Tzovara et al., 2012; Cacioppo et al., 2014; Koenig et al., 2014; Khanna et al., 2015; Mahe et al., 2015). The time window has been determined by testing time-point by time-point, the topographical ANOVA analysis, and microstate classes on momentary grand-mean maps (Koenig et al., 2011). Some recent studies, for example, have explored the most suitable time window from the most fitted microstate maps via the clustering of spatiotemporal ERP by comparing the ERPs of individual subjects with the obtained ERPs of clustering from grand average data (Bailey et al., 2019; Berchio et al., 2019; Ruggeri et al., 2019). Although obtaining global optimal cluster maps by clustering both group (grand average) and individual datasets assigning time-points to template maps is a straightforward solution (Michel and Koenig, 2018), it is challenging to set of template maps from grand average ERP, which reliably represent individual subjects brain responses.

Consensus clustering, as a reliable and stable clustering method, has been successfully used for processing biological data (Monti et al., 2003; Abu-Jamous et al., 2013, 2015a; Liu et al., 2015; Mahini et al., 2017), human brain functional magnetic resonance imaging and EEG data processing (Liu et al., 2017a, b; Song et al., 2019), and multidataset consensus clustering (Filkov and Skiena, 2004; Hoshida et al., 2007; Abu-Jamous et al., 2015b; Liu et al., 2015). However, there has been little discussion about the role of multidataset consensus clustering on individual data from spatiotemporal ERP aimed to identify the ERP components. This is critical because of the difference between the subjects regarding the response time and delay and difference in the quality of recorded data. Therefore, a robust method is required for processing information about the subjects.

The rationale of the current study is to investigate three major points; first, in the ERP experiment, several ERP components are inevitably generated; however, a few of them are targeted, which are more probably elicited if the ERP experiment is run again (Kappenman and Luck, 2012b). Those targeted ERP components are more probably elicited among multiple subjects. The proposed method isolates reliable time windows for ERP of interest for each condition/group. Second, essentially, even after the well-done preprocessing of the collected data, there are still some remaining interferences and some overlapped brain activity with the ERP of interest in the time domain. Therefore, it is practically expected the time window for measuring the amplitude of the ERP of interest includes information of the ERP. One strategy is to check whether the consecutive multiple topographies of time-points are similar or not. If they are similar enough, they come from the same brain activity of the ERP in terms of the linear transformation model of EEG. Thus, such a time window should be determined. Since the time window contains mostly the ERP of interest, the analysis of the brain response can be more accurate. This can result in a better understanding of cognitive processes. Finally, the ERP signal is elicited from numerous similar responses from the subjects. Defining the ERP of interest from the clustering of grand average data neglects the information about individual subjects. Thereby, the new methodology explores the ERP of interest from individual subjects using a multisubject consensus clustering.

In this article, we develop a stabilized multiple-subject consensus clustering (from the multiset consensus clustering family) approach for reliably clustering spatiotemporal ERP data in both individual subjects and group levels. This can provide a novel mechanism to explore the cognitive functions in ERP/EEG data. Furthermore, we use a newly proposed time-window determination method to obtain the most suitable time window for a given ERP of interest. We do expect the new methodology can retrieve the consistent response among the subjects in a group to discover a reliable time window for the ERP of interest. To assess the efficiency and reliability of our method, the proposed method is applied to simulated and the prospective memory experiment data (Chen et al., 2015). The proposed method has been tested to identify two state-of-the-art ERPs, namely, N2 and P3 components in simulated data, and isolating N300 and prospective positivity components in the real data.

## Materials and Methods

This section describes first two ERP datasets including conducted simulated data and real data. Then, our proposed method is described in detail. Finally, two classes of statistical analysis for assessing the studied methods are explained.

### ERP Studies

#### Simulated ERP Data

**FIGURE 1:**
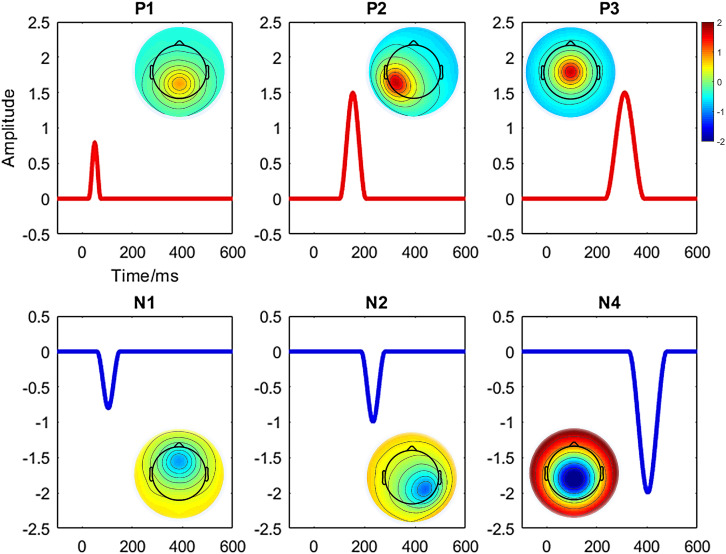
Illustration of topography maps and waveforms for the defined six components (i.e., P1, N1, P2, N2, P3, and N4) in simulated ERP data.

We conducted a simulated ERP data using the BESA dipole simulator^1^ for assessing the performance of the studied clustering methods aimed to identify the predefined ERP components. Entirely, six components (i.e., P1, N1, P2, N2, P3, and N4) and two conditions (i.e., “Cond1” and “Cond2”) from a group of 20 subjects were defined. A simulated scalp with 65 electrodes was used for representing the spatial (i.e., topographic) information. Each trial was epoched from 100-ms prestimulus to 600-ms poststimulus at a sampling rate of 429 Hz. The averaged reference method was used for referencing. The topography maps of the components and corresponding waveforms are shown in Figure 1. Among the defined components, we studied N2 referring to the maximum negative voltage in 201- to 265-ms poststimulus [i.e., it was defined in 175–292 ms via simulator (Figure 1)]. The time window was calculated using the signed area measurement method (Sawaki et al., 2012). Similarly, P3 component refers to the positive response (266–357 ms) poststimulus (i.e., defined in 240–385 ms according to Figure 1). Meanwhile, the signal was manipulated using the MATLAB function *awgn* (i.e., adding white Gaussian noise) to add a reasonable noise (i.e., signal-to-noise ratio = 20 dB) on signal power measured for each simulated dataset as a whole. Furthermore, random movement of two ERPs (e.g., changing the original signal by randomly increasing/decreasing maximum five time-points) was applied to the original signal from the 20 individuals’ data. The electrode sites for measuring statistical amplitude power differences were defined as P6/PO4 and CPz/Cz for N2 and P3, respectively.

#### Real ERP Data

The prospective memory experiment (Chen et al., 2015) data were used as real ERP data to assess the performance of the proposed method. Following the prior study, the experiment data included 20 symptomatically remitted patients, i.e., with schizophrenia (RS) and 20 healthy control (HC) participants. Two tasks, namely, prospective memory (PM) and ongoing task, were investigated. The EEG data were recorded with 32 electrodes (SynAmps amplifier, NeuroScan) and epoched from 200-ms prestimulus to 1,000-ms poststimulus. Furthermore, a 30 Hz (24 dB/octave) digital low-pass filter was applied. Two target ERP components, N300 and prospective positivity components, were studied. The N300 referred to the maximum negative voltage, over the occipital region, hypothetically between 190 and 400 ms, and the prospective positivity represented the maximum positive voltage, over the parietal region, and between 400 and 1,000 ms.

### Proposed Method

**FIGURE 2:**
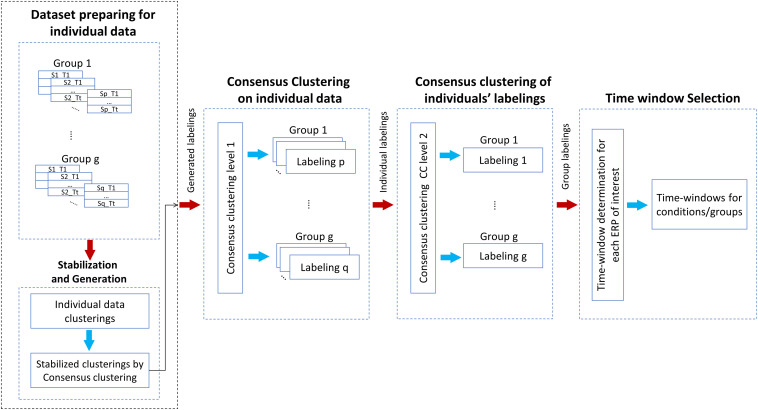
Illustration of the steps of the proposed multisubject consensus clustering for processing the ERPs of interest. The scheme of the proposed method on *g* groups of subjects is demonstrated. S = subject, T = task.

The graphical explanation of the proposed method is illustrated in Figure 2. Besides, Procedure 1 and Procedure 2 are presented for a better representation of the new methodology. Noteworthy to mention that we have employed a mechanism to obtain the optimal number of clusters by, first, running the consensus clustering many times followed by determining the optimal number of clusters based on the quality of obtaining time windows (Mahini et al., 2019). The details of the proposed method are given as follows:


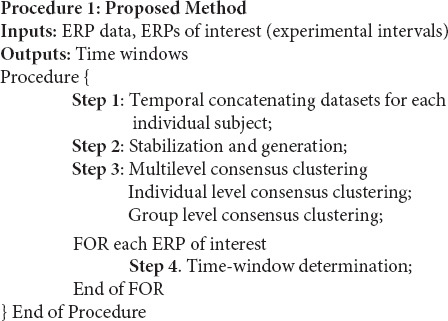


#### Dataset for Clustering

The collected multiple data points by a high-dense EEG sensor array consist of the spatial topographies of brain activities (i.e., each time-point corresponds to a topography). We have investigated the spatiotemporal ERP data where the time-points are clustered based on their topographical similarity. For each subject, a larger dataset was yielded from temporal concatenating (Murray et al., 2008; Calhoun et al., 2009) the associated datasets from all conditions together. For example, given a subject’s ERP data from 300 time-points, 2 conditions, and 65 electrodes, the temporal concatenated dataset with a dimension of 600 × 65 is used for clustering. Therefore, the samples for clustering individual data are the time-points, and the features are represented by the topography (i.e., the electrode field configuration). The goal of clustering is to find the consecutive time-points sharing similar topographies in which the neural responses remain stable for periods of time called time window.

#### Stabilization and Generation

We utilized the cluster-based similarity partitioning algorithm method (Karypis and Kumar, 1998; Nguyen and Caruana, 2007) as the consensus function based on pairwise similarity measurement between partitions. This function was used for each level of consensus clustering and the stabilization step of the proposed method. Before the generation step, two important issues, consensus clustering configuration and stabilized generation, are necessary to be investigated. Several clustering methods were considered for selecting the appropriate configuration of consensus clustering. Hence, *k*-means (Pascual-Marqui et al., 1995; Pena et al., 1999) and hierarchical clustering (Tibshirani and Walther, 2005) with correlation similarity function, fuzzy *c*-means (FCM; Bezdek, 1981), self-organizing maps (SOMs; Kohonen, 1990), diffusion map spectral clustering (Sipola et al., 2013) consisting *k*-means with Euclidean similarity, and modified *k*-means (Pascual-Marqui et al., 1995), and AAHC (Murray et al., 2008) using spatial correlation, were used for the generation purpose. Thereby, for appropriate consensus clustering configuration, modified *k*-means was used as a benchmark [i.e., the accepted clustering method in many studies (Michel and Koenig, 2018)] to be compared with other studied clustering methods. The clustering methods with higher mutual similarities with modified *k*-means in the majority of clustering results of individuals data (e.g., ≥50% of the subjects), were selected using in the generation phase. Rand index (Strehl and Ghosh, 2003; Meila, 2007) was used to measure the mutual similarity between the results of each clustering method on individual data and modified *k*-means. Rand index can be calculated using the following equation:

(1) $$ℛ\left(L,L^{\prime }\right)=\frac{N_{11}+N_{00}}{n\left(n-1\right)/2}$$

where *n* denotes the number of observations and *N*_00_ denotes the number of object pairs in different clusters from both *L* and *L*′ clusterings. While *N*_11_ denotes the number of object pairs in the same clusters in *L* and *L*′.

Additionally, a stabilization procedure based on consensus clustering was designed for the clustering generation of consensus clustering (at the subject level). The stable clustering refers to the clustering results in which the mutual similarity between two or more clustering results is closed to 1 in theory. To measure stability, a mechanism based on the testing similarity of two clustering results was utilized. If they are highly similar, the clustering method is robust. The consensus clustering of grand average ERP data from multiple runs of each stochastic clustering method (e.g., from 2 to 20 repeats that can be changed if necessary) was employed to find the appropriate number of repetitions to get stable clustering. The optimal number of repetitions should satisfy the following two conditions:

(2) $$\max\left(\left|R_{r}-R_{r-1}\right|,\left|R_{r}-R_{r+1}\right|\right)\leq \varepsilon$$

where

$$R_{r}=ℛ\left(L^{*-r},L^{*-\left(r-1\right)}\right)$$

$$L^{*-r}=\arg\max_{L\in {𝕃}_{\bar{X}}}\sum_{r=2}^{Mr}\Gamma \left(L_{r}\right)$$

and

(3) $$\min\left(R_{r-1},R_{r,}R_{r+1}\right)\geq \tau$$

where Γ denotes the consensus function, L^*−*r*^ denotes the consensus clustering results from *r* repetitive results (i.e., maximum repeats denotes by *Mr*) of stochastic clustering method, which is indicated by *L*_*r*_, and $\bar{X}$ denotes the grand average from the individual datasets. Furthermore, *R*_*r*_ denotes the mutual similarity between the consensus clustering results from *r* and *r* − 1 repetitions. Thus, a proper number of repetitions is determined by measuring the mutual similarity among the results of consensus clustering. In other words, the optimal repetition option is selected when the mutual similarity between *r* − 1 and *r*, and between *r* and *r* + 1 reaches a suitable similarity threshold (e.g., τ ≥ 90), and the change among mutual similarities tends to very small values (e.g., ε ≤ 0.03).

#### Multilevel Consensus Clustering

A two-level consensus clustering was utilized for finding the best fitted clustering from individual subjects. The proposed two-level multisubject consensus clustering is explained by the following notations:

Let *S* = {*S*_1_, *S*_2_, …, *S*_*p*_} denotes a set of subjects from a group, and *X* = {*x*_1_, *x*_2_, …, *x*_*n*_} denotes a set of time-points for individual data, in which each time-point *x*_*s*_ = {*e*_1_, *e*_2_, …, *e*_*f*_}, *s* = 1, 2, …, *n* (*f* denotes the number of electrodes) is a vector of features/channels (i.e., it can be represented in the spatial dimension as a topography map). Besides, $L_{i}^{j}=\left\{C_{1,i}^{j},C_{2,i}^{j},\ldots ,C_{k,i}^{j}\right\}$ represents the clustering results for *j*th clustering method *j* = 1, 2, …, *m*, for *i*th subject, *i* = 1, 2, …, *p* with *k* number of clusters. Thus, $C_{w,i}^{j}$ is defined as *w*th cluster, *w* = 1, 2, …, *k* from *j*th method for *i*th subject. The result of the first-level clustering for each of individual datasets is denoted as:

(4) $$L_{i}^{*-opt}=\arg\max_{L\in {𝕃}_{X}}\sum_{j=1}^{m}\Gamma \left(L_{j}^{i}\right)$$

where, $L_{i}^{*-opt}$ denotes the consensus clustering results of *i*th subject from all possible *k-*partitions on *X*. At the second level, another consensus clustering is used on the first level clustering results across the subjects (i.e., in the group level), which is defined as:

(5) $$L^{**-opt}=\arg\max_{L\in {𝕃}_{S}}\sum_{i=1}^{p}\Gamma \left(L_{i}^{*-opt}\right)$$

where, L^**−*opt*^ denotes the result of consensus clustering across the subjects.

Taken as a whole, the optimal ensemble clustering across the subjects can be noted by:

(6) $$L^{**-opt}=\arg\max_{L\in {𝕃}_{X,S}}\sum_{i=1}^{p}\sum_{j=1}^{m}\Gamma \left(L_{j}^{i}\right)$$

To provide a better sense of implementation of the proposed method, the multisubject consensus clustering was implemented in MATLAB platform, as demonstrated in Figure 2 and Procedure 1.

#### Time Window Determination

The time window determination procedure explores the measurement time window by analyzing the temporal and spatial characteristics of the result cluster maps. The inner-similarity of the candidate cluster map (the maps in the experimental measurement area) and their overlapping with the defined experimental time interval, were considered to estimate the proper time windows. First, the inner-similarity of candidate maps is calculated aimed to detect those with the consecutive time-points with a high spatial correlation. The inner-similarity of a cluster map is the mean of correlation coefficients between topography maps of each two different time-points. More in detail, to calculate the inner-similarity of a cluster map, first, the spatial correlation coefficient (Murray et al., 2008; Micah et al., 2009) of time-points was calculated. Therefore *Cor*_*v,u*_ denotes the correlation coefficient between the topographical maps of *u* and *v* as two time-points in the cluster map. Then, for each row, the distance matrix can be calculated as:

(7) $$D_{v}=d\left(Cor_{v,u},Cor_{v,v}\right),u\neq v$$

where, *D* denotes the distance matrix in which each row is calculated by the distance between each element in the row and *Cor*_*v,v*_ (i.e., self-correlation) in correlation matrix (*Cor*). To variance-stabilizing transformation of the calculated correlation, fisher *z*-transform (Fisher, 1921) was used for each vector *D*_*v*_ (i.e., every row of distance matrix) before calculating the mean of the distance matrix *D*_*avg*_. Finally, an inverse *z*-transform of *D*_*avg*_ was used for calculating inner-similarity as shown below:

(8) $$InnSim=1-D_{\text{avg}}$$

Hypothetically, in the ERP component, the spatial correlation between the time-points is close to 1 indicating consecutive time-points that represent a cognitive process. Therefore, among the candidate cluster maps, the cluster maps with higher inner similarity than the threshold (e.g., ≥0.90) were selected for overlap testing. We have selected a realistic choice of 0.9 as a satisfactory threshold for time-window qualification. Next, among those cluster maps, the cluster map with the greatest inner-similarity and overlapping was selected as the best suitable cluster map for representing the time window [i.e., via the properties (start, end, and duration)]. More details for implementing the time-window selection method are presented in Procedure 2.


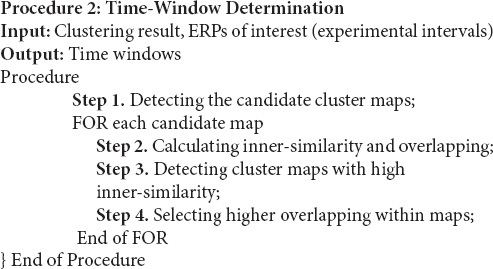


### Statistical Analysis

Two classes of *p* values based statistical measurements were used to evaluate the performance of the proposed method. First, two one-sided tests (TOST; Rogers et al., 1993; Harms and Lakens, 2018) was performed on simulated data to test the similarity between ground truth and estimated time windows by measuring the obtained time-window properties (start, end, and duration). Second, a statistical power analysis was used by employing repeated measures ANOVA for both simulated and real data. Further, for testing the robustness of those methods, the statistical analysis results were calculated on over 50 independent runs of the studied methods. Overall, we tried to assess the meaningfulness, accuracy, and robustness of the proposed methodology.

The TOST test was accomplished by setting equivalence margin [−δδ] in [−5 5] ms (can vary depending on the dataset and quality of discriminability). Two composite null hypotheses tested the assumption of the differences: *H*0_1_: (μ_1_ − μ_2_) ≤ − δ and *H*0_2_: (μ_1_ − μ_2_) ≥ δ, where μ_1_, μ_2_ are the mean of each series in the comparison (e.g., the estimated start points from all the individual subjects in a group and corresponding ground truth start points). When both null hypotheses can be statistically rejected, it can be concluded that the observed effect falls within the equivalence margins and practically equivalent (Seaman and Serlin, 1998). In other words, the difference between the mean of the estimated values and the corresponding ground truth values should not exceed the equivalence margins. Furthermore, a repeated-measures ANOVA for the simulated data with the within-subject factor: task (“Cond1” and “Cond2”) was considered for statistically analyzing N2 component in the electrode sites: P6/PO4 for N2 and CPz/Cz for P3. The test was applied to the mean amplitude of N2 and P3 in the estimated time windows separately. Similarly, the statistical power analysis for real data was carried out via repeated measures ANOVA (i.e., mixed 2 × 2) with the addition of a between-subject factor: group (RS and HC) and the within-subject factor: task (PM and ongoing). The test was applied to the mean amplitude of N300 and prospective positivity. The selection of electrodes was based on prior ERP findings (Chen et al., 2015). Specifically, the amplitude of N300 over the occipital region (electrodes: O1/Oz/O2) and prospective positivity over the parietal region (electrodes: P3/Pz/P4) were measured. Statistical comparisons were made at *p* values of *p* < 0.05 for both data.

## Results

**FIGURE 3:**
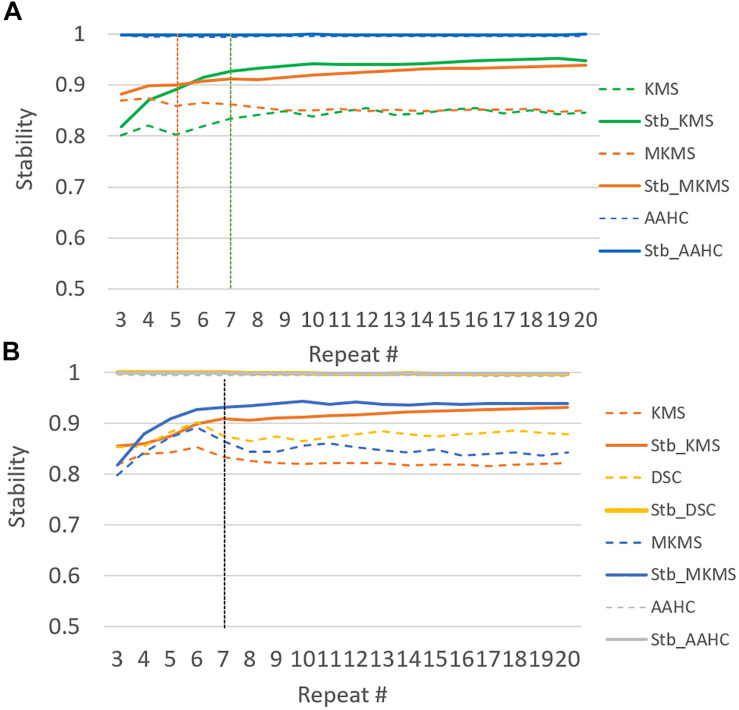
The illustration of the stability test in 20 runs of studied clustering methods to the grand average ERP data. Dash lines demonstrate the original clustering method stability and the continuous lines illustrate the corresponding stabilized version for each studied clustering method behavior for the range of repetition (e.g., from 2 to 20). **(A)** Stabilizing in the simulated data and **(B)** stabilizing in the real data.

To achieve the appropriate clustering result, several important parameters were adjusted, (i) determination of the optimal number of clusters: following our previous study (Mahini et al., 2019), the appropriate number of clusters for simulated and real data was determined in five and six cluster maps, respectively. (ii) The configuration of the proposed consensus clustering: among the studied clustering methods (addressed in “Stabilization and Generation”), *k*-means, hierarchical clustering, AAHC, and modified *k*-means methods were applied to the simulated data. Similarly, *k*-means, FCM, SOMs, diffusion map spectral clustering, AAHC, and modified *k*-means methods were selected for the clustering of real data (Table 1). (iii) Generating stabilized clustering from stochastic clustering methods: following (section “Stabilization and Generation”) the optimal repeat for modified *k*-means and standard *k*-means was obtained in five and seven repeats for the simulated data (Figure 3). Likewise, those clustering methods met stability in seven repetitions in real data. Furthermore, a realistic inner-similarity threshold (e.g., ≥0.90) and a sufficient number of time-points for selecting the candidate cluster maps, e.g., a minimum of 60 to 100 ms (Grieder et al., 2016; Koenig and Brandeis, 2016) were determined.

**TABLE 1:** The illustration of the clustering method selection by calculating the similarity of the results with the modified *k*-means method for individual data.

Data	Group	KMS	HC	FCM	SOM	DSC	AAHC
**Simulated data**	**G1**	**19**	**14**	0	0	0	**20**
**Real data**	**RS**	**19**	9	**17**	**17**	**15**	**20**
	**HC**	**19**	11	**19**	**19**	**15**	**18**

*The marked methods with bold font are selected where they achieved higher similarity (rand index) for the majority of individual data (e.g., ≥50% of subjects and similarity ≥ 0.7). RS, remitted schizophrenia; HC, healthy control; G1, simulated group; KMS, k-means; HC, hierarchical clustering; FCM, fuzzy c-means; SOM, self-organizing map; DSC, diffusion maps spectral clustering; and AAHC, atomize and agglomerate hierarchical clustering.*

### Results of Simulated ERP Data

**FIGURE 4:**
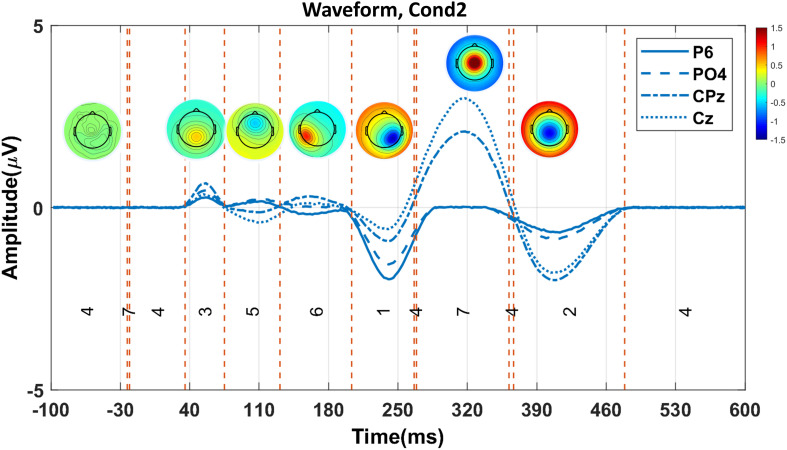
The proposed consensus clustering results in the simulated Cond2 data. Red and blue colors indicate positive and negative potential values, respectively. Each topography map represents a cluster map. P1, N1, P2, N2, P3, and N4 components are presented with cluster maps 3, 5, 6, 1, 7, and 2, respectively. Note that cluster map 4 does not show any component in the simulated data.

We applied the proposed consensus clustering in the simulated data aimed to illustrate all the predefined ERP components. The clustering in seven cluster maps successfully isolated all predefined six components (Figure 4) P1, N1, P2, N2, P3, and N4 correspond with the cluster maps 3, 5, 6, 1, 7, and 2, respectively. Note that cluster map 4 refers to the brain state before stimulus onset and does not present any predefined ERP component.

#### Time Windows and Topographies for ERPs of Interest

**FIGURE 5:**
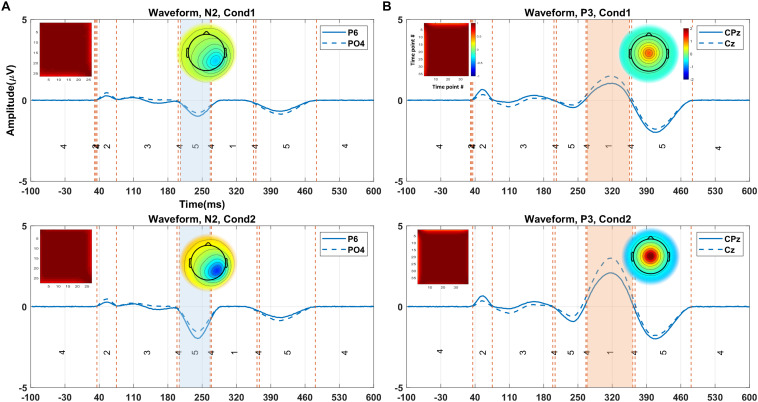
The illustration of the clustering results (shown in the grand average data) for eliciting N2 and P3, including the corresponded topography maps and the spatial correlation of time-points, obtained by the proposed method on the simulated data. **(A)** Selected time window identified by cluster map 5 (i.e., blue area from 205 to 264 ms) for N2 in Cond1 **(upper panel)**. The selected time window by cluster map 5 (i.e., orange area from 203 to 264 ms) for N2 in Cond2 **(lower panel)**. **(B)** Selected time window with cluster map 1 (i.e., colored area from 264 to 350 ms) for P3 in Cond 1 **(upper panel)**. Selected time window identified by cluster map 1 (i.e., colored area from 268 to 357 ms), the topographic map for P3 in Cond 2 **(lower panel)**. The range of the color bars is equally associated with the plot sections. Cond1 = condition 1, Cond2 = condition 2.

Figure 5 illustrates the clustering results and the elicited N2 and P3 components (from one random execution), including the corresponded topography maps and the spatial correlation of time-points obtained by the proposed method on the simulated data. Figure 5A indicates that the N2 component in Cond1 and Cond2 are elicited by cluster maps 5 (marked blue). Likewise, Figure 5B illustrates that the P3 component is identified by the microstate map 1 (marked orange) in both conditions. These results reveal that a significant main effect of task (*p* < 0.0001) was identified in N2 in the duration of microstate maps. Similarly, a significant main effect of task (*p* < 0.0001) was detected in the P3 component. For both components, the measured amplitudes were greater in Cond2. This reveals that the N2 and P3 components seem to be distinctly elicited by the proposed method in the simulated data.

#### Comparison Between Estimated and Ground Truth Time Windows

**FIGURE 6:**
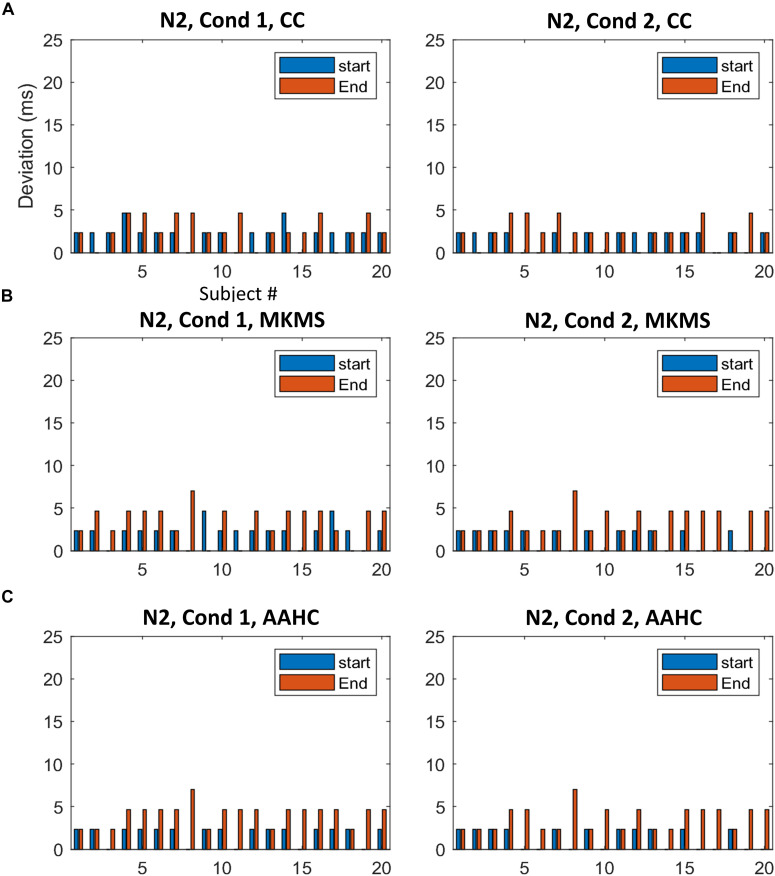
The difference between the estimated time windows from corresponding ground truth time windows (i.e., based on start and end criteria) for individual data in N2. **(A)** The estimation results of the proposed method. **(B)** The estimation results by MKMS method. **(C)** The estimation results by AAHC method. MKMS, modified *k*-means; AAHC, atomize, and agglomerate hierarchical clustering.

**FIGURE 7:**
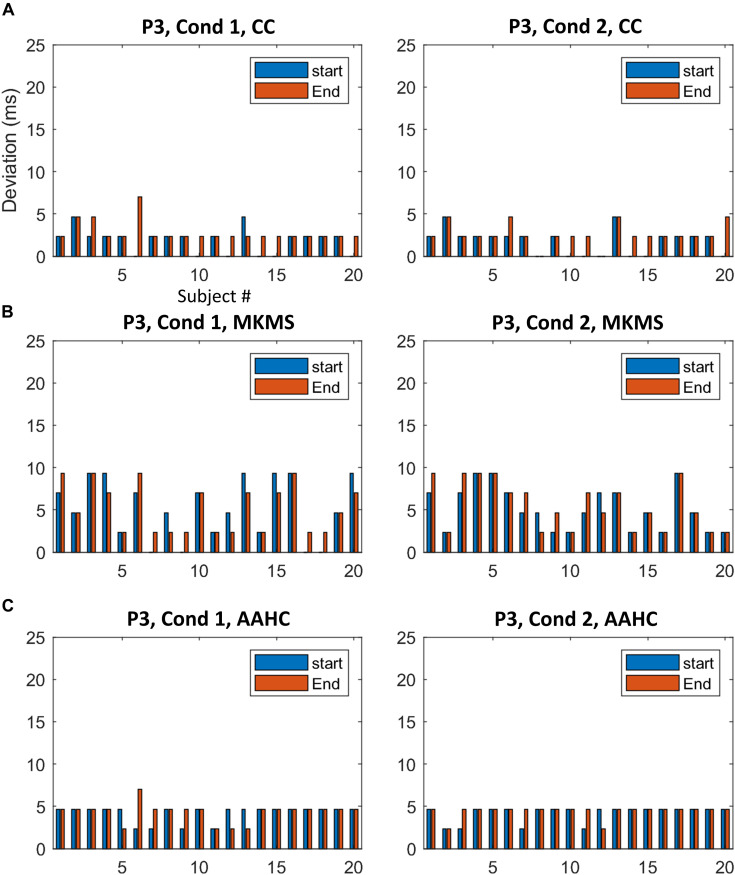
The difference between the estimated time windows from corresponding ground truth time windows for individual data in P3. **(A)** The results from the proposed method. **(B)** The estimation results by MKMS method. **(C)** The estimation results by AAHC method.

The proposed method was compared with the state-of-the-art clustering methods, namely, modified *k*-means and AAHC, in spatiotemporal ERP clustering. Our time-window selection method was applied to the clustering results (i.e., proposed consensus clustering, modified *k*-means, and AAHC results) to identify each ERP of interest. The Start, End, and Duration parameters of estimated time windows were compared with that of the ground truth time windows (obtained from the simulation) on the clustering results of individual data for testing the accuracy. The TOST result (Table 2) for N2 component from clustering methods illustrates that the null hypothesis was rejected for the proposed method, modified *k*-means, and AAHC for all parameters in both conditions except End in Cond1 for AAHC. Similarly, the null hypothesis was rejected in all parameters except Duration in both conditions for the proposed method. It was, however, not rejected in either of the criteria in P3 for modified *k*-means and AAHC. Taken as a whole, the proposed method achieved a more precise estimation of time windows in individual data. Moreover, for a better sense of comparison between studied clustering methods, the accuracy of estimated (i.e., based on Start and End parameters) time windows for the subjects is exhibited in Figures 6, 7. It is observable that the consensus clustering method outperforms modified *k*-means and AAHC in terms of accuracy of estimation, especially in P3 component.

**TABLE 2:** Descriptive two one-sided tests (TOST) equivalence tests between ground truth TWs (time windows) and estimated TWs by the proposed consensus clustering (CC), modified *k*-means (MKMS), and atomize and agglomerate hierarchical clustering (AAHC) in individual subjects’ data from simulated ERP data.

Comp-Meth	Cond	Criteria	*p1*	*p2*	DiffMu (ms)	EQ_interval (ms)	
**N2_CC**	C1	Start	0.000	0.003	2.2	0.4	4.1
		End	0.000	0.003	2.6	1.0	4.1
		Duration	0.000	0.000	0.4	–1.6	2.3
	C2	Start	0.000	0.001	1.6	–0.4	3.6
		End	0.000	0.002	2.3	0.7	4.0
		Duration	0.000	0.000	0.7	–1.2	2.6
**P3_CC**	C1	Start	0.000	0.000	1.9	0.5	3.2
		End	0.018	0.000	2.8	–4.7	–0.9
		Duration	**0.380**	0.000	4.7	–6.8	–2.6
	C2	Start	0.000	0.000	1.8	0.3	3.2
		End	0.008	0.000	2.6	–4.4	–0.7
		Duration	**0.266**	0.000	4.3	–6.4	–2.2
**N2_MKMS**	C1	Start	0.000	0.001	2.1	0.3	3.9
		End	0.000	0.041	3.5	1.9	5.1
		Duration	0.000	0.000	1.4	–0.3	3.1
	C2	Start	0.000	0.001	1.4	–0.6	3.4
		End	0.000	0.030	3.5	2.0	5.0
		Duration	0.000	0.001	2.1	0.5	3.7
**P3_MKMS**	C1	Start	0.000	**0.543**	5.1	2.8	7.5
		End	**0.548**	0.000	5.1	–7.3	–3.0
		Duration	**0.997**	0.000	10.3	–13.8	–6.7
	C2	Start	0.000	**0.554**	5.1	3.2	7.0
		End	**0.654**	0.000	5.5	–7.8	–3.1
		Duration	**0.999**	0.000	10.6	–14.0	–7.3
**N2_AAHC**	C1	Start	0.000	0.001	1.8	0.0	3.5
		End	0.000	**0.104**	4.1	2.7	5.5
		Duration	0.000	0.000	2.3	0.9	3.7
	C2	Start	0.000	0.000	1.3	–0.6	3.2
		End	0.000	0.039	3.6	2.1	5.1
		Duration	0.000	0.001	2.3	0.8	3.9
**P3_AAHC**	C1	Start	0.000	**0.162**	4.2	2.7	5.7
		End	**0.244**	0.000	4.3	–6.2	–2.4
		Duration	**0.999**	0.000	8.5	–10.6	–6.4
	C2	Start	0.000	**0.167**	4.2	2.6	5.8
		End	**0.276**	0.000	4.4	–6.3	–2.6
		Duration	**0.999**	0.000	8.6	–10.7	–6.6

*Bold marked represent nonsignificant results. Comp-Meth, component of interest and the method; Cond, condition; C1, condition 1; C2, condition 2; p1, p value of lower bound; p2, p value of upper bound; DiffMu, difference of mean of two sets; and EQ_interval, confident equivalence interval.*

### Results of Real ERP Data

#### Time Windows and Topographies for ERPs of Interest

**FIGURE 8:**
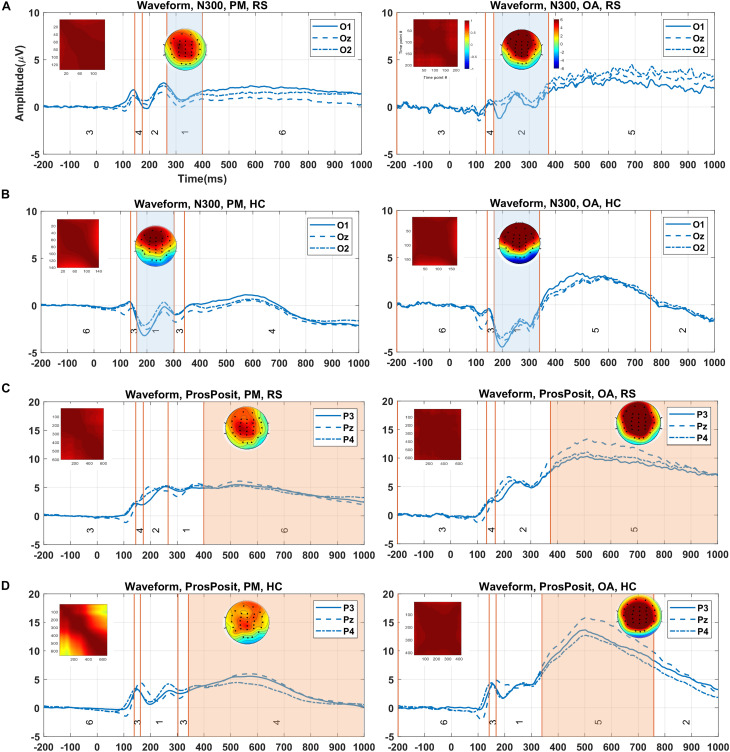
Demonstration of the clustering result (showed in the grand average data), identified time window for ERP of interest, and corresponding topography map and spatial correlation of the time-points in each group/condition via the proposed method. **(A)** Identified time window by cluster maps 1 and 2 (i.e., colored areas) for two tasks (PM and ongoing) for N300 in RS group. **(B)** Selected time windows identified with map 1 for both conditions in the HC group. **(C)** The isolated time windows by cluster maps 6 and 5 for the tasks (PM and ongoing) in RS group. **(D)** Equally, the time windows identified by cluster maps 4 and 5 for the tasks (PM and ongoing) in the HC group. The visual comparison between two groups in panels **(A,B)** for N300 and in panels **(C**,**D)** for prospective positivity shows the difference in the waveforms in the selected time windows. The color bars are equally associated with the plot sections. PM, prospective memory; OA, ongoing task; Pros.Pos, prospective positivity; RS, remitted schizophrenia; HC, healthy control.

The clustering results (randomly selected) from running the proposed method on real data for N300 and prospective positivity components, the corresponding topography maps, and the spatial correlation of time-points are illustrated in Figure 8. N300 identified by the cluster maps 1 and 2 in the RS group, is illustrated by the colored area in Figure 8A for both PM and ongoing tasks. Furthermore, N300 identified by cluster map 1 in the HC group and two tasks (PM and ongoing), is illustrated in Figure 8B. Similarly, the prospective positivity component is isolated by the cluster maps 6 and 5 in the RS group for PM and ongoing tasks, respectively (Figure 8C). The identified prospective positivity by cluster maps 4 and 5 in the HC group for PM and ongoing tasks are illustrated, respectively (Figure 8D). The average topographies shown in Figure 8 are obtained from the selected time windows identified by the cluster maps. Hence, the statistical power analysis revealed that HC was characterized by a more negative potential over the occipital-central electrodes (*p* < 0.001). Additionally, a silently larger positive potential was localized over frontal-central electrodes compared to the RS group in N300. Moreover, a slightly more negative potential was observed over occipital-central electrodes (*p* < 0.001) in the ongoing task from both RS and HC groups in the N300 component. Our results revealed no significant difference for prospective positivity regarding group factor; however, a larger positive potential was localized over central electrodes (*p* < 0.0001) in the ongoing task comparing to the PM task.

#### Statistical Analysis and Stability Test Results

The mean *p* value and standard deviation (SD) were obtained from over 50 independent runs of the studied clustering methods and statistical analysis on the individual data (Table 3). Rendering to stability analysis, the proposed method (SD = 0.003) was more stable compared to modified *k*-means (SD = 0.006) for the main effect of group and less stable than AAHC (SD = 0.002) for N300 component. Interestingly, it was the most stable method compared to other studied clustering methods for both the main effect of task (SD = 0.002) and interaction between group and task (SD = 0.043). Besides, the statistical power analysis results showed that the main effects of group and task by the proposed method were significant (*p* < 0.002 for both factors). Likewise, the main effect of group was significant by the modified *k*-means (*p* < 0.017) and AAHC (*p* < 0.004). The main effect of task, however, was significant only via AAHC (*p* < 0.013). Meanwhile, the interaction between group and task was not significant in both modified *k*-means and AAHC. Similarly, the proposed method was statistically the most stable for the interaction between group and task (SD = 0.011) comparing to other studied clustering methods in prospective positivity (Table 3). Additionally, the main effect of task was significant (*p* < 0.0001), and, more importantly, the interaction between group and task was also significant (*p* < 0.007) by the proposed method. However, the main effect of group was not significant by the proposed method. The main effect of task was also significant by modified *k*-means (*p* < 0.0001) and AAHC (*p* < 0.0001), whereas, the main effect in group, and the interactions between group and task were not significant by both modified *k*-means and AAHC methods.

**TABLE 3:** Mean *p* value and standard deviation (SD) calculations of statistical power analysis results in over 50 runs of study clustering methods on the individual data for the real data.

	N300			Pros.Pos.		
Method	Group	Task	intGrTsk	Group	Task	intGrTsk
Proposed (*p* value)	**0.002**	**0.002**	0.058	0.590	**0.000**	**0.007**
SD	0.003	**0.002**	**0.043**	0.227	0.000	**0.011**
MKMS(*p* value)	**0.017**	0.101	0.303	0.614	**0.000**	0.150
SD	0.006	0.075	0.225	0.199	0.000	0.156
AAHC (*p* value)	**0.004**	**0.013**	0.145	0.662	**0.000**	0.246
SD	**0.002**	0.009	0.133	0.201	0.000	0.131

*IntGrTsk, interaction between group and task; Pros.Pos., prospective positivity. Bold marked represent significant results.*

## Discussion

This study proposed a new methodology based on multisubject consensus clustering on spatiotemporal ERP data for the suitable time-window determination. To this end, we designed the stabilized multisubject consensus clustering in two levels described as follows: (i) subject resolution in which the stabilized consensus clustering was used to combine the results of various clusterings on each subject’s data in the group; (ii) group resolution in which the most suitable clustering for each group was obtained by consensus clustering of the clustering results of individual data. From the ERP technique point of view, the researchers using the ERP technique for the cognitive neuroscience research often face up the challenge to determine a time window for an ERP, since the most popular textbook of ERP recommends the readers averaging the amplitudes in the time window as the measurement of the ERP peak amplitude (Luck, 2014). In terms of previous publications, we found that the determination of such a time window has mostly relied on the visual inspection, which can be subjective and bring bias to conclusions and difficulty for the readers to repeat the experiment. Therefore, the main objective of this work was to provide a reliable clustering-based mechanism (objective approach) for studying the temporal dynamic and sensory information about the subjects (i.e., brain responses). This was accomplished with the multilevel clustering mechanism and the time-window determination method. The clustering result from entire subjects entails important information about group response which is critical for studying the cognitive processes in ERP.

One issue in processing individual data is, apart from the need for sufficient trials for obtaining reliable ERP (Boudewyn et al., 2018) and the variety of brain responses in the trials, the variability associated with individual subjects’ brain responses, which is observable when ERPs are used to assess cognitive functions. The underlying assumption is that the variety in the trials and subject responses are involved in ERP, although in the ERP techniques, the assumption is that the ERP is phase-locked and time-locked. Therefore, each subject grants value to the statistical test in terms of differences between conditions or groups, which is through the variance across subjects assisting in the ability to detect a significant experimental effect (Kappenman and Luck, 2012a). Yet, in the literature, the individual responses were mostly addressed by fitting the cluster maps of individual data to the cluster maps of group average data (Murray et al., 2008; Koenig et al., 2014; Michel and Koenig, 2018; Berchio et al., 2019; Ruggeri et al., 2019). To cover this gap, we strived to cluster individual subject data in the first level and map the entire individual clusterings into a group as the ultimate clustering.

From the cluster analysis view of point, the various clustering strategies such as using the single clustering method on the different types of datasets; repeated clustering with a single clustering method and combining the results; and the multiple-clustering methods applied to the individual dataset potentially affect the clustering quality (Abu-Jamous et al., 2013, 2015b; Liu et al., 2015; von Wegner et al., 2018). To investigate this issue and reliably feeding consensus clustering, two data-driven based mechanisms were appropriated before multilevel cluster analysis. First, consensus clustering configuration was performed aim to find the appropriate clustering methods. This was recognized by calculating the similarity between candidate clustering methods and modified *k*-means (benchmark) from individual data. Second, the stabilized clusterings were carried out by stabilizing the stochastic clusterings. Taken as a whole, these two procedures can make an additional sense of obtaining reliable and stable results instead of using a single clustering method or the conventional consensus clustering platform. Noteworthy to mention that clustering selection and stabilization can result in different configurations for various ERP data.

In accordance with the obtained results, two major differences were noticed between the proposed method and conventional clustering methods:

(i) The statistical test in this study revealed that the proposed method estimates a more precise time windows for individual subjects in comparison with the other conventional clustering methods in simulated data for both ERPs of interest (N2 and P3). The foremost reason is that our method uses the strength of multiple clustering methods and data-driven processing individual subject data to fit the suitable time windows for each condition/group, despite with using spatial consistency comparison between ERPs of individual and grand average data (Habermann et al., 2018; Michel and Koenig, 2018; Berchio et al., 2019).
(ii) According to the statistical analysis results (Table 3), the proposed method outperformed other benchmark methods regarding achieving more stability in the real data. Over 50 independent runs of the clustering on the same datasets, the estimation of the proposed method was with a much smaller variance. This indicates that the estimation of the ERP time window was much closer to the ground truth time window of the ERP, in contrast to the other methods. Such results from the real data also correspond to the ones from the simulation data, i.e., the estimation of the time window of an ERP was more accurate by the proposed method. Therefore, the results of the current study, based on analyzing the brain dynamics from the stimuli onset to the brain response, successfully explored the attention effect on the neural responses from the subjects in real ERP data.

The drawback of the proposed method, however, is that if the real ERP component is still embedded in ERP waveforms the determination of the time window of an ERP component cannot be precise. Indeed, this also happens in the visual inspection method to determine the time window of an ERP. Therefore, in order to determine the time window of an ERP component more precisely, the EEG preprocessing is very critical. The better the preprocessing is, the more precise and objective determination of the time window of an EPR is carried out in terms of the proposed method.

The results of analyzing the brain dynamic responses revealed that the brain electrical dynamics in obtained time windows were comparatively different in time-window properties (start, end, and duration) for different conditions/groups. Therefore, from the clinical point of view, the brain responses from two groups (RS and HC) to the stimuli onset were investigated to identify N300 and prospective positivity components. This can be interpreted as the fact of the variety of brain response for the subjects in different condition/group. In N300 component isolation, for example, the difference was shown in cluster maps 1 (i.e., between RS and HC groups) in PM tasks. Likewise, the duration differed in cluster maps 1 and 2 in the ongoing task between the groups. Again, at the source level, a silently larger negative response was observed in ongoing than PM task in both RS and HC groups. These results demonstrate that RS patients with schizophrenia showed a functional recovery of PM cue detection during the event-based PM task. Consequently, the electrophysiological data revealed the ability of symptomatically remitted patients with schizophrenia to distinguish the PM task from the ongoing task. This was reflected by the significant main effect of task type among these two groups. As a result, this finding showed a complementary viewpoint to the prior studies (Fukumoto et al., 2014; Chen et al., 2015). Our results can be employed for interpreting the advantage of the treatment in RS patients in terms of measuring/identifying the difference in ERPs of interest in the observations. Therefore, this may indicate a degree of functional recovery of preparatory attentional processes that helps the processing of PM task in these subjects (RS patients) during clinical remission. Thus, providing further evidence for the recent researches demonstrating symptomatic remission in schizophrenia is associated with a degree of functional recovery of attentional processes.

## Conclusion and Future Works

This work presents a multisubject consensus clustering technique to explore spatiotemporal ERP by extracting group-level information from individual responses. Our proposed methodology has successfully extended the previous research findings (Murray et al., 2008; Koenig et al., 2014; Michel and Koenig, 2018) of cluster analysis of EEG/ERP. Noteworthy to mention that we have proposed the multiset consensus clustering method in the present study which can work better for the group-level analysis. Since the proposed method is not limited to just ERP data, it is very interesting to apply the proposed method on other brain imaging modalities for investigating the various types of brain dynamics. Furthermore, the proposed method can also be used as an appropriate tool to analyze the single-trial EEG by considering suitable roles for the trials in higher resolution (single-trials) in the future. Taken together, this work emphasizes that, in the time-window determination from spatiotemporal ERP, the temporal dynamics can be extremely influenced through the measurement interval. It is noteworthy that this methodology can be investigated on different levels (i.e., groups, subjects, trials). The current study also highlights that the obtained time windows are sensitive to the responses from the subjects, which can provide a better sense of understanding in information processing of the neural responses. In order to show the effectiveness of the proposed method, we have used the simulated ERP dataset and the real ERP dataset. Indeed, the selection of the real ERP dataset does not mean that the proposed method only works for such an attention-related ERP experiment. The proposed method has no limitation on the experiment types of ERPs. Thereby, a toolbox has been developed under the MATLAB platform, named ERP_CC.^2^ Taken as a whole, we can rely on the information retrieved by the new method, which reflects the attention mechanism regarding the response to the stimuli in the real data. We therefore believe that the EEG neuroimaging method can be studied by the proposed methodology in various dimensions to accomplish useful results in cognitive neuroscience studies.

## Data Availability Statement

The datasets analyzed in this article are not publicly available. Requests to access the datasets should be directed to GC.

## Ethics Statement

The studies involving human participants were reviewed and approved by the ethics committee of PLA general hospital. The patients/participants provided their written informed consent to participate in this study.

## Author Contributions

RM designed the methodology, implemented the algorithms, performed data analysis, produced tables and figures, and wrote the manuscript. YL performed statistical analysis, collected real data, and analyzed the results. WD collected the real data. RF prepared the simulated data. AN designed the methodology and contributed to the final manuscript. GC designed the study (real data), collected real data, and analyzed the results. FY designed the study, analyzed the results, and wrote the manuscript. All authors contributed to the article and approved the submitted version.

## Conflict of Interest

The authors declare that the research was conducted in the absence of any commercial or financial relationships that could be construed as a potential conflict of interest.

## Footnotes

1 https://www.besa.de/products/besa-simulator/besa-simulator-overview/

2 https://doi.org/10.5281/zenodo.3541576
